# Assessing Prognostic Factors in Hodgkin's Lymphoma: Multistate Illness-Death Model

**Published:** 2018-01-01

**Authors:** Fatemeh Javanmardi, Amal Saki-Malehi, Ahmad Ahmadzadeh, Fakher Rahim

**Affiliations:** 1 Department of Epidemiology and Biostatistics, School of Health, Ahvaz Jundishapur University of Medical Sciences, Ahvaz, Iran; 2 Health Research Institute, Thalassemia and Hemoglobinopathies Research Center, Ahvaz Jundishapur University of Medical Sciences, Ahvaz, Iran

**Keywords:** Hodgkin's lymphoma, Multistate model, Prognostic factors, Markov illness-death model

## Abstract

**Background:** Hodgkin's lymphoma (HL) is a unique cancer of lymphocytes that has unknown reason. As lymphocytes are found throughout the lymphatic system, HL can start almost anywhere in the body. It usually starts in a group of lymph nodes in one part of the body; it usually spreads in a predictable form, from one group of lymph nodes to the next. Eventually, it can spread to almost any tissue or organ in the body through the lymphatic system or the bloodstream. So it's important to evaluate the prognostic factors of mortality and recurrence. The aim of this study is to use multistate model to consider the event history of patients and assess important prognostic factors.

**Materials and Methods**: We performed a retrospective review on 389 patients with Hodgkin's disease referred to the Oncology and Hematology Center, Shafa Hospital, Ahvaz during 2002 and 2012. An illness – death model was fitted to assess the hazard of transitions during the course of the disease for each prognostic factor.

**Results:** The results showed that the prevalence rate was higher in male population ≥50 years of age with a hemoglobin level of less than 10.5 g per deciliter and diagnosis of advanced stage of disease. The risk of death for males was twice more than females (HR=2.07). Moreover, patients with mediastina and spleen involvement were more than others in danger of death (1.66 and 1.36, respectively).

**Conclusion:** In conclusion, the multistate model offers an appropriate method to consider the event history of patients and determine main prognostic factors, which play an important role in rapid diagnosis and choosing the best treatment choice for each patient.

## Introduction

 Hodgkin's lymphoma (HL), is a cancer of the lymphatic system that occurs when lymphocytes become cancerous^   1 ^. It usually involves cervical, axillary and inguinal nodes^   2 ^. Hodgkin's lymphoma may occur at any age, but mostly it has been seen in people between ages 15 to 34 and in people over the age of 55 years^   3 ^. It is potentially curable in early stages and significant improvements have been seen in survival rate^   4 ^. Chemotherapy and radiation therapy are two main methods of treatment, and if both methods are used together more desirable results will be achieved. The most important complication of Hodgkin's lymphoma is secondary cancer that appears ten years after initial treatment^   5 ^. The most common secondary cancers are breast cancer, lung, digestive system and sometimes leukemia^   3 ^. In general, Hodgkin's lymphoma patients can experience different and more than one type of event in the disease process^6^. In such situations, separate survival analyses are not making sense since they fail to describe the relations between different types of the endpoints in the disease process^     7 ^. However, multi-state models (MSM) can provide an efficient and convenient statistical analysis for this problem. Multi-state models are useful approach to describe movements of patients between different states such as disease-free status, recurrence, metastasis, secondary cancer and death^     8 ^. These models can estimate transition probability between states, predict the probability of being in next state and hazard of transitions. Another important aspect of MSM is the possibility of prediction of clinical prognosis in patients at a certain point of illness or recovery process, and also determining transition hazard between states for each risk factors^   9 ^. A commonly multi-state model is disability model that includes 3 states and also known as an illness-death model. It is useful for the progressive disease that has forward and irreversible movements and diseases causing increased risk of death^   10 ^. According to this model, the effect of time-dependent variables in the model can consider as an intermediate state. The aim of this study was to use multistate model to consider the event history of patients and evaluate important prognostic factors to assess their effect on each transition during the patient’s history.

## MATERIALS AND METHODS

 This retrospective study that was conducted on 389 patients with Hodgkin's lymphoma referred to Shafa Oncology and Hematology Center in Ahvaz (in the southwest of Iran) during 2002 and 2012. Laboratory data for each patient were collected, and the final status of patients in terms of death or recurrence was registered. The data included initial information such as demographic data, relapse, histologic types, stage of the tumor, treatment protocol, aspiration, lymph node group or organ involved at presentation and the morphological diagnosis of HL (nodal sites involvement). Relapse was identified based on clinical signs or periodic computed tomography (CT) after a period of at least 30 days. Patients whose disease confirmed based on the decision of two pathologists were included in the study and excluded if their files and information were not completed. Cases were staged clinically according to the Ann Arbor staging system. In this study, a Markov illness-death model was used for patients with HL^   10 ^. The Markov assumption implies that progression rate of patients to the next state is independent from their progression rate into the previous state. 

Although, different states for patients may occur in HL progression, relapse and death are more important clinically. However, as the number of states and possible transitions increases, the model will be more complex. 

**Figure 1:**
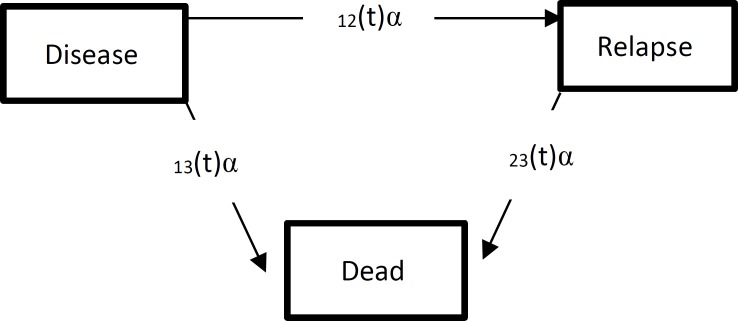
The three-state model for patients with Hodgkin’s lymphoma

In this study, patients are considered to move between three states; disease (1), relapse (2), and dead as the absorbing state. The arrows in Figure 1 indicate the direction of possible transitions. During the study, patients may die straightly (1→3), become worse and experience relapse (1→2) or death after relapse (2→3). The function below represents the hazard rates for moving from state h to state j (or transition intensity rates) at times t and is defined as follows:

α_hj_(t) = $\underset{∆t\to 0}{\lim}\frac{1}{∆t}$ P (patients move from state h to state j in (t,t+∆t|h at t) ) (10)

The entire transition hazards and hazard ratio for some variables such as gender, age, location of lymph, stage, histology (Nodular lymphocyte predominant Hodgkin's Lymphoma, and Classical Hodgkin's Lymphoma), hemoglobin, aspiration (Removal by suction of fluid and cells through a needle) and nodal sites involvement (NSI) of cervical, mediastina, spleen, and axillary were estimated; in fact nodal sites were involved more than other areas. A classical descending method was used to choose prognostic factors in two models. This backward or descending elimination approach begins by calculating hazard ratio and its confidence interval in a model which includes all of the independent variables. Then the variables which were not significant at the 0.05 level were deleted from the model. The AIC criterion was applied to compare the diﬀerent models. The multi-state modeling was done using the R statistical *software* 3.2.3 and (msm) package. This study was approved by Ethics Committee of Ahvaz Jundishapur University of Medical Science (IRAJUMS.REC.1395.488). 

## Results

 The study included 389 patients with a mean age of 27.5 and standard deviation of 15.83 years. 52 patients (13.36%) were 15 years old or less, 241 (61.95%) were between 15 and 34 years, 70 were (18%) between 34 – 55 years, and 26 (6.69%) were over 55 years old. 

The median follow-up was 5.66 years. The population studied consisted of 161 (41.4%) females and 228 (58.6%) males. 6.20% had stage IV disease, 24.7 % had stage III, 21.1% and 21.9% had stage I and II, respectively. 

Based on histologic types, 32 (25.7%) of 289 (74.3%) patients with classical HL had Nodular lymphocyte predominant HL. According to pathology test, all patients were categorized as follows: 164 (42.2%) patients with nodular sclerosing subtype, of whom 94 (24.20%) had mixed cellularity, 21 (5.40%) had lymph node metastases and 2 (0.5%) showed lymphocyte depletion. 

Most of the patients were treated by an ABVD regimen (87.9%) and 12.1% underwent Stanford regimen. Of whom, 57.6 % were diagnosed with cervical involvement and 8% had axillary nodes. Inguinal nodes were reported in 6.4%. Involvement of other parts of body was also reported in 15.2% of study participants. Characteristic of patients are shown in Table1.

**Table1:** Characteristics of Study Patient

**Prognostic factors**	**Frequency**	**Percent**
Sex		
Male	228	58.6
Female	161	41.4
Age groups		
< 15 years	52	13.36
15 – 34 years	241	61.95
34 – 55 years	70	18
> 55	26	6.69
Stage		
I	82	21.1
II	85	21.9
III	96	24.7
IV	24	6.20
missing	102	26.22
location lymph		
Axillary nodes	31	7.96
Inguinal nodes	75	19.28
Cervical nodes	224	57.59
Other part	59	15.17
Histologic types		
Classical HL	289	74.3
Nodular lymphocyte predominant HL	32	25.7
Missing	68	17.48
Classic Variety		
Nodular sclerosing	164	42.15
Mixed cellularity	94	24.17
Lymph rich	21	5.5
Lymphocyte depleted	2	0.5
Missing	108	27.77

During the study, 226 of 389 patients remained in the state of disease, and 135 patients experienced relapse. 23 of whom died without recurrence and 30 patients died after relapse. By the end of study, 99 patients remained in state 2. Table 2 provides the frequencies of pairs of consecutive states.

**Table 2:** Summary of the number of transitions for each state

**Status**	**1 (Disease)**	**2 (Relapse)**	**3 (Death)**
1(Disease)	226 (58.09%)	136 (34.96%)	23(5.91)
2(Relapse)	-	99 (25.44)	30 (7.71)

Table (3) shows the results of the illness-death Markov model for full model. AIC in this model was estimated 825.62. The risk of death for males with HL was twice more than females (HR= 2.07; 95% CI: 0.20-20.60). The results of the present study for different age groups showed that the risk of death after relapse of disease (2→3) in patients within the age range of 15-34 years was 1.5 times (95 % CI: 0.55- 4.12) more than those under 15 years. The hazard ratio of death without relapse was 3.5 times for patients over 55 years in comparison with patients under 15 years. According to histologic diagnosis, patients with axillary involvement nodes were 2.86 (95% CI: 1.07, 7.67) times more likely to die of relapse compared to patients with cervical cancerous lymph nodes. 

The risk of death directly (1→3) in patients with inguinal involvement was 2.73 times more than those with cervical cancerous lymph nodes. Hazard ratio for other parts of the body involvement in transition 2 to 3 was 1.91 times more than cervical cancerous lymph nodes, and hazard of death after relapse for patients in stage II toward stage I was 2.93 with 95% CI (0.43, 19.77). Hazard of relapse for patients in stage III was diploid in comparison with stage I (HR=2.01; 95% CI: 1.08, 3.76).

**Table3:** Prognostic factors for each transition with hazard ratios and their 95% conﬁdence interval in full model

**Variables**		**1→2**			**1→3**			**2→3**		
		**HR**	**CI 95%**	**P**	**HR**	**CI 95%**	**P**	**HR**	**CI 95%**	**P**
Sex	Male	0.99	(0.68,1.42)	0.95	2.07	(0.2,20.6)	0.54	1.24	(0.6,2.55)	0.56
Age	15 - 34	1.37	(0.88,2.14)	0.16	1.29	(0.17,9.46)	0.81	1.51	(0.55,4.12)	0.42
	34 - 55	2.03	(1.20,3.43)	0.008	1.5	(0.10,20.98)	0.76	1.96	(0.66,5.8)	0.22
	> 55	0.52	(0.18,1.46)	0.22	3.51	(0.41,30.14)	0.25	1.48	(0.15,13.9)	0.73
location lymph	Axillary nodes	0.92	(0.5,1.69)	0.78	0.69	(0.01,28.33)	0.86	2.86	(1.07,7.67)	0.03
	Inguinal nodes	0.47	(0.205,1.11)	0.07	2.73	(0.48,15.35)	0.25	0.9	(0.1,7.71)	0.92
	Other	1.26	(0.79,2)	0.33	0.14	(2.19e-09,1.2e+07)	0.83	1.91	(0.83,4.41)	0.12
Stage	II	1.7	(0.88,3.27)	0.11	0.54	(0.01,18.48)	0.76	2.93	(0.43,19.77)	0.27
	III	2.01	(1.08,3.76)	0.02	0.75	(0.04,12.91)	0.84	1.06	(0.15,7.36)	0.95
	IV	2.72	(1.26,5.89)	0.01	1.62	(0.07,36.36)	0.76	2.49	(0.33,18.66)	0.37
NSI Cervical	Yes	1.09	(0.72,1.69)	0.68	0.38	(0.07,2.04)	0.26	0.84	(0.35,1.96)	0.69
NSI Mediastina	Yes	1.03	(0.72,1.46)	0.87	-	-		1.66	(0.85,3.20)	0.13
NSI Spleen	Yes	1.33	(0.9,1.96)	0.15	0.9	(0.12,6.66)	0.91	1.36	(0.67,2.77)	0.39
NSI Paraaortic	Yes	1.96	(1.33,2.94)	<0.001	0.89	(0.06,14.28)	0.93	0.41	(0.18,0.95)	0.03
NSI Axillary	Yes	1.74	(1.20,2.52)	<0.001	-	-		0.77	(0.38,1.58)	0.47
Histology	Nodular predominant	0.51	(0.24,1.05)	0.08	4.04	(0.18,89.86)	0.37	0.59	(0.13,2.64)	0.49
Hemoglobin	< 10.5	1.36	(0.96,1.91)	0.08	10.96	(0.21,571.70)	0.23	1.58	(0.78,3.17)	0.20
Aspiration	Yes	0.65	(0.4,1.06)	0.06	0.41	(0.05,3.22)	0.40	1.20	(0.51,2.85)	0.68

Reference category: Sex: female, Age: less than 15years, Location: cervical nodes, Stage: I, NSI Cervical: No, NSI Mediastina: No, NSI Spleen: No, NSI Paraaortic: No, NSI Axillary: No, Histology: Classical, Hemoglobin: >10.5, Aspiration: No, P‐values less than 0.05 were considered as the level of significance. NSI: Nodal sites involvement according to morphologic diagnosis - means no transition was seen.

 more than classical subtype. Moreover, hazard of relapse for patients with cervical lymph nodes involvement was the same as patients without this complication (HR=1.09; 95%CI: 0.72, 1.69). Patients with mediastina and spleen involvement were more at risk of death than others (1.66 and 1.36, respectively). Recurrence risk for patients with positive axillary and para-aortic involvement was more serious than the patients without these symptoms and hazard ratio was computed 1.96 and 1.74, respectively. Risk of death for patients with hemoglobin level under 10.5 was 10 times more than others, and risk of death after recurrence for patients who had bone marrow involvement was 1.20 times more than those without this complication.

**Table 4:** The results of reduced model in which significant covariates were included. Lower AIC in this model shows better results (AIC =440.24)

		**1→2**			**1→3**			**2→3**		
		**HR**	**CI 95%**	**P**	**HR**	**CI 95%**	**P**	**HR**	**CI 95%**	**P**
Age	15 - 34	1.83	(1.14,2.93)	0.01	1.11	(0.44-2.8)	0.82	1.38	(0.77,2.47)	0.28
	34 - 55	2.76	(1.11,6.86)	0.02	1.83	(0.32,10.46)	0.50	2.3	(1.24,4.26)	<0.001
	> 55	0.76	(0.33,1.75)	0.51	3.83	(0.89,16.48)	0.07	1.76	(0.37,8.37)	0.47
location lymph	Axillary nodes	1.66	(1.08,2.55)	0.02	0.83	(0.12, 1.71)	0.85	3.23	(1.24,8.30)	0.01
	Inguinal nodes	0.41	(0.18,0.93)	0.03	3.64	(0.36,36.80)	0.27	1.82	(1.06,3.12)	0.03
	Other	1.37	(0.68,2.76)	0.37	0.31	(2.42e-05,3971.07)	0.8	1.73	(0.91,3.28)	0.09
Stage	II	1.12	(0.96,1.30)	0.14	0.71	(0.12,4.20)	0.70	3.21	(0.76,13.55)	0.11
	III	2.41	(1.17,4.96)	0.01	0.96	(0.16,5.76)	0.96	1.74	(0.43,7.04)	0.43
	IV	3.5	(1.8,6.8)	<0.001	1.98	(1.1,3.49)	0.01	3.21	(0.64,16.1)	0.15
NSI Paraaortic	Yes	2.83	(1.45,5.52)	<0.001	0.93	(0.54,1.60)	0.7	0.62	(0.32,1.20)	0.15
NSI Axillary	Yes	2.3	(1.62,3.26)	<0.001	-	-	-	0.64	(0.53,0.77)	<0.001

Reference category: Age: less than 15 years, Location lymph: cervical nodes, Stage: NSI Paraaortic: No, NSI Axillary: No; NSI: Nodal sites involvement according to morphologic diagnosis

The estimated hazard ratios from reduced model in Table 4 shows involvement in axillary and inguinal nodes increase the risk of relapse and death after that (1→2 and 2→3). The rate of death in patients between 34 and 55 years old increases after relapse. So, death hazard is 2.3 more than in patients less than 15 years. A significant association was seen between high clinical stage and rate of death. Patients in stage IV were 1.98 times *more* likely to die in comparison with stage I of disease. 

**Figure2:**
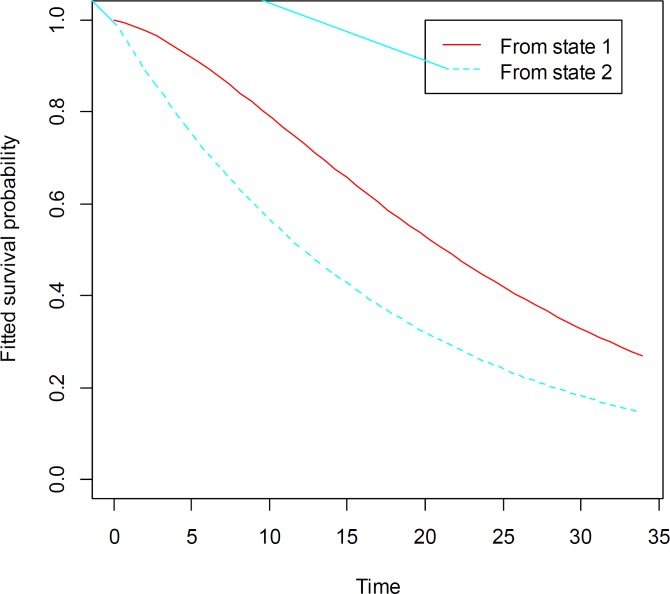
Estimated survival probability over time (months) from each nonabsorbing state to death among HL patients

The estimated survival curves for each of transitions are plotted in Figure 2. This shows patients with relapse were *more* likely to have died in comparison with patients in primary disease state. The 10-month survival probability for patients with relapse is close to 0.6, as opposed to 0.8 for patients in state 1. In overall, disease relapse may decrease the probability of survival. 

## Discussion

 One of the main goals in oncology is to determine the effect of prognostic factors at each stage of the disease. Understanding the nature of cancer and the factors affecting it can cause lifestyle changes, improvement of life quality and treatment response. Prognostic factors can be considered in the early diagnosis and treatment can extend the life of these patients. In addition to the prognostic factors, intermediate events effect on patient’s survival, but standard statistical analyses are unable to investigate all disease pathway. Recently, multi- state models have become popular and are frequently used for clinical research, especially on cancer ^7^^,^^11^^,^^12^ . Here, according to The International Prognostic Score (IPS), some factors that predict which Hodgkin’s lymphoma patients are at high risk for relapse and death are discussed as the results of full model (i.e. stage, hemoglobin, age, sex). Some factors may not be statistically significant, but they are clinically important and effect on disease process. 

Similar to previous studies, the current study showed that patients with relapse experience were at high risk risk of death^   13 ^. In fact, the incidence relapse is associated with worse survival from the time of diagnosis. Although there are a number of clinical and laboratory factors associated with an increased risk of relapse, the causes of relapse are not yet clearly defined. Prognostic factors of Hodgkin’s lymphoma can be helpful in the decision making process.

 Age is one of the most important factors for determining survival of patients. Epidemiological studies of HL have shown that that age distribution is bimodal peaking in second decade of life and after age 50^   14 ^^.^ According to our results, the risk of relapse and death will increase by age. As aging make older people more susceptible to diseases, chemotherapy has more effective impact on young patients^   14 ^^.^ A comparison between the elderly patients and their young counterparts have shown that the presence of EBV in neoplastic cells and high frequency of mixed cellularity histologic types can affect the development of HL^   15 ^. Increasing latently infected B cell in the elderly, leading an increased risk of positive EBV in HL patients (16). In one study conducted on classical HL, an adverse effect of EBV- positive on overall survival was observed among patients between 50 and 74 years old defined as subgroup. In fact, it was considered as a prognostic factor, especially in the elderly patients^   17 ^. In another study in the United Kingdom, an adverse association between disease-specific survival (DSS) and positive-EBV was also observed in patients over 60 years ^     18 ^. Another study on patients with HL showed that old age (over 50 years) is one of the determinants of prognosis, and elderly patients are at high risk of recurrence and death. It was also revealed that old age is associated with short survival^   3 ^. According to the International Prognostic Score (IPS), the most widely risk stratiﬁcation index for HL, the identified risk factor for HC included age over 45 years^   19 ^**.(check it)** But some studies concluded that age is not a significant factor for survival of HL patients^   4 ^. 

Gender plays an important role as a prognostic factor. Based on the results of current study, Hodgkin's lymphoma with less survival occurred more often among men. Results of one retrospective study showed^3^ that follicular lymphoma and diffuse large B-cell lymphoma are the reasons for better survival in females compare to their male counterparts. Like our study, Kermani et al.(2005) found that the incidence of Hodgkin's lymphoma is more than twice as common among men as among women (HR=2). The same result was also obtained in another study^   20 ^^.^ IPS and some other studies have not considered sex as an effective factor^21^^-^^29^. The results of this study and others ^   20 ^ have indicated that there is a significant negative association between low red blood cell count (hemoglobin level below 10.5 g/dl) and survival. A significant reduction in hemoglobin level was observed in patients with advanced disease, systemic (B) symptoms and aggressive histological subtypes^   30 ^. However, investigators from Birmingham^   31 ^ have not seen any difference between patients with hemoglobin level under 12 g/dl and upper 12 g/dl. The most important clinical risk factor for HL patients is stage of disease^   31 ^. Similar to previous studies, we concluded that stage III and IV are bad prognostic factors and increase hazard rate for death^   4 ^. In fact, higher stage increases the risk of recurrence among patients^   3 ^. In other words, stage IV is more dangerous than others^   20 ^. In some studies, overall survival for late stage-disease( III / IV) was significantly lower than that for the other stages (I / II)^   22 ^. But some studies have shown the stage of disease as an ineffective variable^   21 ^. A common method used to investigate the relationship between different states of disease and effect of risk factors is MSM indicating the progress of morbidity during the specified time period^     8 ^. In this study, a three illness-death model was used to evaluate the risk factors of patients with HL. MSM described time of transitions between states. These more complex models can be represented by description of various intermediate events and computing the probability of survival and hazard for each transition. The multistate model has been introduced for evaluating transition intensity or hazard rate^   32 ^. An interesting aspect of these hazard ratios is the possibility of comparing these rates between the different endpoints^     7 ^. Assessment of risk factors in this study showed that gender, age, stage of disease and level of hemoglobin are bad prognostic factors for HL disease. It is worth noting that the location of lymph node involvement, considered as a substantial covariate resulted from the second model in current study, has not already been discussed. Future studies are required to investigate in more details the role of risk factors in the incidence of HL. 

## CONCLUSION

 In conclusion, the multistate model offers a proper way to identify the prognostic factors playing an important role in rapid diagnosis and choosing the best treatment strategy for this disease. Despite its many limitations, this approach cannot be ignored. In order to estimate accurate coefficients for each transition and each covariate, a further study with a larger samplesize is recommended. 
